# Volumetric and Voxel-Wise Analysis of Dominant Intraprostatic Lesions on Multiparametric MRI

**DOI:** 10.3389/fonc.2019.00616

**Published:** 2019-07-05

**Authors:** Joon Lee, Eric Carver, Aharon Feldman, Milan V. Pantelic, Mohamed Elshaikh, Ning Wen

**Affiliations:** ^1^Department of Radiation Oncology, Henry Ford Health System, Detroit, MI, United States; ^2^Department of Radiology, Henry Ford Health System, Detroit, MI, United States

**Keywords:** prostate cancer, multiparametric MR, dominant intraprostatic lesions, tumor delineation, radiotherapy

## Abstract

**Introduction:** Multiparametric MR imaging (mpMRI) has shown promising results in the diagnosis and localization of prostate cancer. Furthermore, mpMRI may play an important role in identifying the dominant intraprostatic lesion (DIL) for radiotherapy boost. We sought to investigate the level of correlation between dominant tumor foci contoured on various mpMRI sequences.

**Methods:** mpMRI data from 90 patients with MR-guided biopsy-proven prostate cancer were obtained from the SPIE-AAPM-NCI Prostate MR Classification Challenge. Each case consisted of T2-weighted (T2W), apparent diffusion coefficient (ADC), and K^trans^ images computed from dynamic contrast-enhanced sequences. All image sets were rigidly co-registered, and the dominant tumor foci were identified and contoured for each MRI sequence. Hausdorff distance (HD), mean distance to agreement (MDA), and Dice and Jaccard coefficients were calculated between the contours for each pair of MRI sequences (i.e., T2 vs. ADC, T2 vs. K^trans^, and ADC vs. K^trans^). The voxel wise spearman correlation was also obtained between these image pairs.

**Results:** The DILs were located in the anterior fibromuscular stroma, central zone, peripheral zone, and transition zone in 35.2, 5.6, 32.4, and 25.4% of patients, respectively. Gleason grade groups 1–5 represented 29.6, 40.8, 15.5, and 14.1% of the study population, respectively (with group grades 4 and 5 analyzed together). The mean contour volumes for the T2W images, and the ADC and K^trans^ maps were 2.14 ± 2.1, 2.22 ± 2.2, and 1.84 ± 1.5 mL, respectively. K^trans^ values were indistinguishable between cancerous regions and the rest of prostatic regions for 19 patients. The Dice coefficient and Jaccard index were 0.74 ± 0.13, 0.60 ± 0.15 for T2W-ADC and 0.61 ± 0.16, 0.46 ± 0.16 for T2W-K^trans^. The voxel-based Spearman correlations were 0.20 ± 0.20 for T2W-ADC and 0.13 ± 0.25 for T2W-K^trans^.

**Conclusions:** The DIL contoured on T2W images had a high level of agreement with those contoured on ADC maps, but there was little to no quantitative correlation of these results with tumor location and Gleason grade group. Technical hurdles are yet to be solved for precision radiotherapy to target the DILs based on physiological imaging. A Boolean sum volume (BSV) incorporating all available MR sequences may be reasonable in delineating the DIL boost volume.

## Introduction

Prostate cancer is the most common malignancy of men in the U.S., with an annual incidence of 161,360 cases resulting in 26,730 deaths (1). Most patients are diagnosed with disease localized to the prostate, for which radiation therapy is an important curative treatment modality.

In the modern era of dose-escalated radiation therapy, the entire prostate gland is treated to the same dose of radiation irrespective of the biopsy-proven region of disease. Multiple randomized studies have demonstrated that dose-escalation improves biochemical progression-free survival (2, 3). It has also been reported that local recurrences are dose-dependent and most frequently occur at the site of the dominant intraprostatic lesion (DIL) (4, 5)—defined as the most prominent cancerous lesion within the prostate which also exhibits the most aggressive clinical behavior. Numerous studies have suggested that the addition of a boost to the DIL is safe and efficacious without increased acute or late toxicity (6–14).

Multiparametric magnetic resonance imaging (mpMRI) is rapidly becoming the standard diagnostic imaging modality for prostate cancer. mpMRI can be defined as any functional form of MR imaging which supplements standard anatomical T1- (T1W) and T2-weighted (T2W) MR sequences. Namely, this includes diffusion-weighted imaging (DWI), which measures the Brownian motion of water molecules in tissue; dynamic contrast-enhanced (DCE) sequences, which assess tumor angiogenesis and detect microvascular vessel wall permeability; and MR spectroscopy (MRS), which analyzes the chemical composition of prostatic tissue, and compares it to that of cancerous tissue.

mpMRI has shown potential to increase the accuracy of tumor detection, localization, and characterization of prostate cancer (15–18). It has been demonstrated to have a negative predictive value of up to 95% for clinically significant prostate cancer (defined as the presence of Gleason pattern 4 or greater) (19, 20). Whole amount histopathology has been used a gold standard reference to evaluate DIL detection and localization accuracy using mpMRI (21).

The correlation of tumor volume defined by pathology and mpMRI was also investigated and it showed strong dependence on both imaging techniques and specimen processing workflow (22). There are still lack of studies to investigate whether a specific MR sequence is optimal or if a combination of MR sequences is mandatory in accurately delineating the DIL for radiotherapy planning. In this study, we performed volumetric and voxel-wise analyses of tumor foci delineated in three MR sequences and report the level of concordance between them. Furthermore, we quantitatively correlated these results with tumor location and Gleason grade group.

## Materials and Methods

Robust mpMRI data from 90 patients with MRI-guided biopsy-proven prostate cancer were obtained from the SPIE-AAPM-NCI Prostate MR Classification Challenge (23, 24). All images were acquired using two different types of Siemens 3-Tesla MR scanners (the Magnetom Trio and Skyra) without an endorectal coil. Each dataset consisted of T2W, ADC, and volume transfer coefficient (K^trans^) images computed from DCE sequences.

The T2W images were acquired using a turbo spin echo sequence (TE/TR: 5,660/104 ms, Flip Angle: 90° with image resolution of 0.5 × 0.5 × 3.0 mm^3^). The DWI was acquired with a single-shot echo planar imaging sequence with diffusion-encoding gradients in three directions (TR/TE: 2,700/63 ms, with image resolution of 2.0 × 2.0 × 3.0 mm^3^). The ADC map was calculated from three b-values of 50, 400, and 800 s/mm^2^.The DCE series were acquired using a 3-D turbo flash gradient echo sequence (TR/TE: 3.4/1.5 ms, with image resolution of 1.5 × 1.5 × 3.0 mm^3^ and a temporal resolution of 3.5 s). The standard Tofts model was used for pharmacokinetic modeling of the contrast concentration curves. An automated reference tissue method was used to estimate the arterial input function (25). The transfer constant (K^trans^) parametric maps were calculated from the contrast concentration curves.

**Figure 1:**
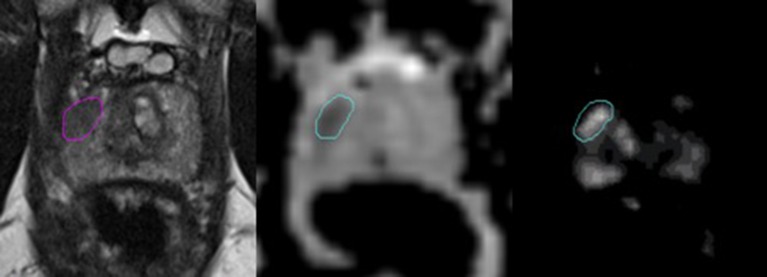
Representative images of a dominant intraprostatic lesion contoured on an T2W image, ADC map, and K^trans^ map (from left to right). The axial T2W images were carefully scrutinized along with those in the coronal, and sagittal planes (not shown) to confirm the presence of a hypodense lesion corresponding to the location of the DIL annotated by an experienced radiologist. In the axial T2W image above, a hypointense lesion is demonstrated in the right peripheral zone. This same area was then assessed for values that were lower and higher than the surrounding normal prostate tissue in the rigidly-registered axial ADC and K^trans^ maps, respectively. Of note, areas of hypervascularity outside of the contoured region in the K^trans^ map shown above were assumed to represent normal vasculature within the central zone of the prostate gland, as a corresponding area suspicious for cancer was not visualized in either the T2W image or the ADC map.

An experienced radiologist annotated suspicious lesions on each MR modality, and MRI-guided biopsies were performed to confirm the aggressiveness of the disease (i.e., Gleason grade grouping). The tissue specimens were examined by expert pathologists and the results were defined as the ground truth in this study. Both the ADC and K^trans^ image sets were rigidly co-registered and resampled using linear interpolation to match those of the T2W images. For example, resampling transformed the resolution from 2.0 × 2.0 × 3.0 mm (ADC) and 1.5 × 1.5 × 3.0 mm (K^trans^) to 0.5 × 0.5 × 3.0 mm (T2W). The intraprostatic lesions were then identified and contoured on each MR sequence separately for every patient by a radiation oncologist based on the radiologist's annotation following criteria of hypointense values on the T2W images (window 718, level 360) and ADC maps (window 3,000, level 1,500) and high values on the K^trans^ maps (window 39, level 21). The DIL was separately contoured by a second radiation oncologist for a subset of MR images (19 patients) to assess for interobserver variability. Representative images of an intraprostatic lesion contoured on an ADC map, K^trans^ map and T2W image are shown in Figure 1.

The anatomic location of the intraprostatic lesions as well as their corresponding Gleason grade group (1–5) were available for each patient. Due to the small number of data points available, Gleason grade groups 4 and 5 were analyzed together. To evaluate the quantitative correlation between contours on each imaging modality and its statistical dependence on tumor location and Gleason grade group, the 95 percentiles of Hausdorff distance (HD), mean distance to agreement (MDA), Dice coefficient, and Jaccard index were calculated between the contours for each pair of MR sequences (i.e., T2W vs. ADC, T2W vs. K^trans^, and ADC vs. K^trans^). These variables are defined in Table 1.

**Table 1:** Definitions of contour evaluation metrics.

**Definitions**
Hausdorff distance (HD): The distance from one point in a subset to the closest point in another subset.
*d*_*H*_ = max[sup inf *d*(*x, y*), sup inf *d*(*x, y*)]
Mean distance to agreement (MDA): The average of the Hausdorff distances within a defined metric space.
Dice coefficient: Measure of the degree of overlap between sample sets, with a value of 1.0 representing complete overlap (range: 0–1.0).
$DICE=\frac{2*A\bar{\cap }B}{A+B}$
Jaccard index: Comparison of the similarity and diversity of sample sets, with a value of 1.0 representing unity (range 0–1.0).
$Jaccard=\frac{A\bar{\cap }B}{A+B-A\bar{\cap }B}$

For the voxel-wise analysis, a Boolean sum volume (BSV) was defined as a combination of the contours from all three image modalities for each patient. This additional step was performed to ensure that an equal number of representative voxels from each MR sequence were included in the analysis. Fractional ranks were then obtained for each voxel of the BSV and the Spearman correlation was calculated. It is worth noting that the Spearman correlation was selected because a monotonic relationship was assumed between each pair of contours, as opposed to a linear relationship in which case a Pearson correlation may have been more appropriate.

## Results

The DILs were located in the anterior fibromuscular stroma, central zone, peripheral zone, and transition zone in 35.2, 5.6, 32.4, and 25.4% of patients, respectively. Gleason grade groups 1–5 represented 30.3, 39.4, 17.2, and 13.1% of the study population, respectively (with group grades 4 and 5 analyzed together). The mean contour volumes for the T2W images, and the ADC and K^trans^ maps were 2.14 ± 2.1, 2.22 ± 2.2, and 1.84 ± 1.5 mL, respectively. K^trans^ values were indistinguishable between cancerous regions and normal prostatic tissue for 19 patients.

The Dice coefficient and Jaccard index were 0.74 ± 0.13, 0.60 ± 0.15 for T2W-ADC, and 0.61 ± 0.16, 0.46 ± 0.16 for T2W-K^trans^. For the voxel-based portion of the study, the Spearman correlations were 0.20 ± 0.20 for T2W-ADC and 0.13 ± 0.25 for T2W-K^trans^.

Tables 2, 3 summarize the Spearman correlation, Dice coefficient, Jaccard index, as well as the HD and MDA by DIL location and Gleason grade groups, respectively.

**Table 2:** Hausdorff distance (95%), Mean distance to agreement, Dice coefficient, Jaccard index, and Spearman-rank order by tumor location.

**Location**		**AFS**	**Peripheral**	**Cent/Tran**	**Total**
T2-ADC	HD	4.46 ± 1.69	4.11 ± 1.39	4.36 ± 2.43	4.32 ± 1.89
	MDA	0.96 ± 0.53	0.94 ± 0.45	1.00 ± 0.77	0.97 ± 0.60
	Dice	0.73 ± 0.12	0.69 ± 0.16	0.74 ± 0.16	0.74 ± 0.13
	Jaccard	0.59 ± 0.14	0.55 ± 0.17	0.61 ± 0.18	0.60 ± 0.15
	Spearman	0.15 ± 0.16	0.26 ± 0.26	0.18 ± 0.13	0.20 ± 0.20
T2-K^trans^	HD	6.46 ± 2.42	5.77 ± 2.82	6.57 ± 4.18	6.21 ± 3.25
	MDA	1.65 ± 0.65	1.53 ± 0.97	1.63 ± 1.13	1.59 ± 0.95
	Dice	0.61 ± 0.15	0.59 ± 0.17	0.61 ± 0.19	0.61 ± 0.16
	Jaccard	0.45 ± 0.16	0.44 ± 0.17	0.46 ± 0.18	0.46 ± 0.16
	Spearman	0.18 ± 0.22	0.06 ± 0.26	0.19 ± 0.27	0.13 ± 0.25

*Cent/Tran, central/transition; AFS, anterior fibromuscular stroma*.

**Table 3:** Hausdorff distance (95%), Mean distance to agreement, Dice coefficient, Jaccard index, and Spearman-rank order by Gleason grade group.

**Gleason grade group**		**1**	**2**	**3**	**4, 5**
T2-ADC	HD	4.33 ± 2.24	4.21 ± 1.96	4.20 ± 1.26	4.61 ± 1.28
	MDA	0.94 ± 0.68	0.96 ± 0.61	0.94 ± 0.37	1.03 ± 0.57
	Dice	0.73 ± 0.15	0.73 ± 0.13	0.71 ± 0.14	0.70 ± 0.21
	Jaccard	0.59 ± 0.15	0.59 ± 0.15	0.56 ± 0.15	0.57 ± 0.22
	Spearman	0.21 ± 0.16	0.25 ± 0.20	0.20 ± 0.19	0.04 ± 0.19
T2-K^trans^	HD	6.07 ± 3.53	5.89 ± 2.87	6.07 ± 2.44	7.51 ± 4.62
	MDA	1.65 ± 1.29	1.50 ± 0.68	1.40 ± 0.66	2.02 ± 1.33
	Dice	0.60 ± 0.22	0.60 ± 0.14	0.65 ± 0.12	0.57 ± 0.22
	Jaccard	0.46 ± 0.21	0.44 ± 0.15	0.50 ± 0.12	0.43 ± 0.22
	Spearman	0.15 ± 0.24	0.10 ± 0.23	0.12 ± 0.32	0.15 ± 0.27

**Figure 2:**
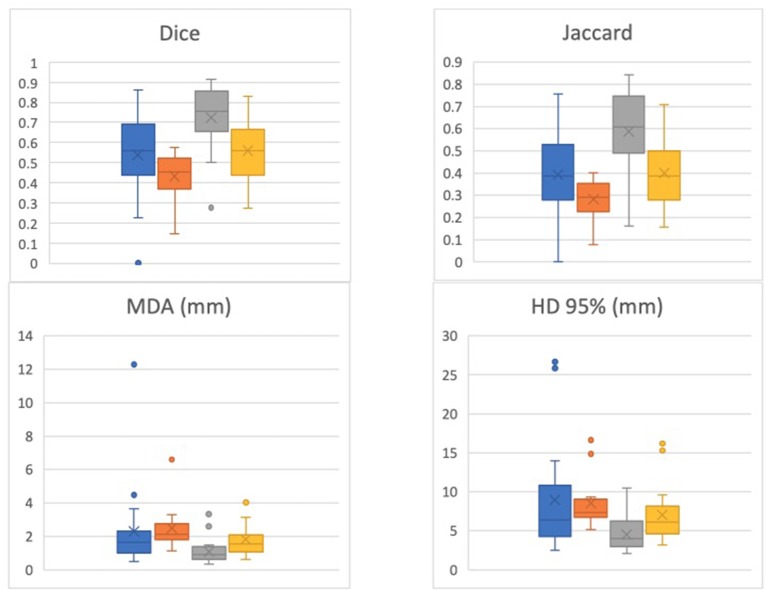
Dice, Jaccard, Mean Distance to Agreement (MDA), and 95% Hausdoff Distance (HD) for 19 patients contoured by the two different physicians. One physician's contour comparisons (i.e., T2-ADC vs. T2-K^trans^) are denoted by blue and orange, respectively, while the other physician's contour comparisons (i.e., T2-ADC vs. T2-K^trans^) are denoted by gray and yellow, respectively. The data suggests strong dependence on physician performance and relatively high interobserver variability.

The DIL was separately contoured for 19 patients by a second radiation oncologist to assess for interobserver variability (these results were not analyzed with respect to Gleason Grade and location). For this second set of contours, the Dice coefficient was 0.51 ± 0.19 for T2W-ADC and 0.42 ± 0.13 for T2W-K^trans^. A comparison between the Dice and Jaccard coefficients, MDA, and HD for the 19 patients contoured by the two different physicians is shown in Figure 2.

Table 4 shows the mean, minimum, and maximum pixel values within the ADC and K^trans^ contours of the 90 patients split according to the Gleason grade group. The results of grade groups 4 and 5 were combined together due to the smaller sample sizes. The mean ADC contoured pixel values ranged from 964.14 to 1007.74 mm^2^/s among different groups while the K^trans^ values ranged from 3.27 to 6.21 min^−1^.

**Table 4:** Mean, minimum, and maximum contoured pixel values for ADC and K^trans^.

**Gleason grade group**	**Number of patients (%)**	**ADC Mean**	**ADC min**	**ADC max**	**ADC *SD***	**K^**trans**^ mean**	**K^**trans**^ min**	**K^**trans**^ max**	**K^**trans**^*SD***
		**mm/s**	**mm/s**	**mm/s**		**Min^**−1**^**	**Min^**−1**^**	**Min^**−1**^**	
1	30.3	964.14	661.55	1247.63	136.70	3.27	0	13.54	3.52
2	39.4	988.43	822.13	1380.78	130.65	3.81	0	10.54	3.19
3	17.2	1007.74	656.63	1334.02	163.89	6.21	0	17.29	4.92
4+5	13.1	999.62	770.11	1806.99	251.47	3.56	0	16.28	5.37

*SD, standard deviation*.

## Discussion

Current national guidelines recommend dose-escalated radiation therapy to the entire intact prostate gland for men receiving definitive radiation therapy for prostate cancer. This approach has demonstrated clear benefits in biochemical progression-free survival across multiple randomized controlled studies. Unfortunately, biochemical recurrence rates can exceed 25% at 10 years necessitating further salvage therapy, which can adversely affect quality of life. Up to 90% of local recurrences after conventional radiation therapy have been shown to occur at the site of the DIL (4, 26). This coupled with the tremendous technological advancements in diagnostic imaging (27) and modern radiation therapy techniques such as treatment under image-guidance (28–30), has led to emerging interest in more accurately targeting the intraprostatic lesion and delivering a further boost to the dominant site of disease.

While the majority of these efforts have been realized using intensity-modulated radiation therapy (7, 9, 11, 31–33), studies have also included dose-escalation using brachytherapy with biologically equivalent doses of around 200 Gy to the DIL (6, 34–36), and more recently with stereotactic body radiotherapy using a simultaneous integrated boost technique (14, 32, 37, 38).

### Clinical Outcomes of Radiation Therapy Boost to the DIL

Early results have demonstrated efficacy with low acute and late toxicities with either treatment approach. A recent systematic review of dose-escalated radiation therapy to the DIL demonstrated that the average grade 3+ gastrointestinal and genitourinary late toxicity was ~2–3% for intensity-modulated radiation therapy, 6–10% for stereotactic body radiotherapy, and 2–6% for brachytherapy (39). The median 5-years biochemical progression-free survival was reported to be 85%. However, the study population included patients of all risk groups with heterogenous use of androgen deprivation therapy. These factors need to be taken into consideration when interpreting the results of these studies.

### mpMRI as a Tool for Target Volume Delineation

mpMRI demonstrates high sensitivity and specificity in the diagnosis and staging of prostate cancer, and its utility in this context has been extensively investigated (40, 41). However, its use as a tool in target volume delineation for the purposes of radiation treatments has not been adequately elucidated. Several barriers exist to incorporating mpMRI to define adequate radiation treatment volumes, one of the most significant being a lack of sufficient data to determine which mpMRI sequence is most accurate in defining the DIL. Groenendaal et al. developed a logistic regression model on DCE and DWI images to predict tumor presence and validated on whole-mount section histopathological images for 12 patients. The model achieved a receiver operating characteristic curve of 0.70 (41). However, the study was limited to peripheral zone and low Gleason score lesions (6 and 7). In addition, each image modality reflects different biological characteristics and may be individually inconsistent in tumor delineation particularly at the voxel level. It is unclear whether a combination of MR sequences would confer any advantage compared to a single mpMRI sequence when contouring the DIL, especially with respect to clinical outcomes such as biochemical control.

Considering the technical variability and lack of consensus on ADC and K^trans^ values for intraprostatic lesions, we did not use automatic threshold values for segmentation. ADC and K^trans^ values can have significant variations across different scanning protocols and MR scanners which makes quantitative analysis difficult. The monoexponential ADC model was used to describe the water diffusion behavior in this study. The b value selection as well as the duration and strength of diffusion sensitizing gradients could have impact on the ADC value. And the ADC values depended on many factors including cell density, size, shape, permeability, and perfusion effects. The complex diffusion dynamics of biological tissue required more advanced compartment models such as intravoxel incoherent motion and vascular, extracellular, and restricted diffusion for cytometry in tumors. It was difficult to achieve an optimal balance of spatial and temporal resolution of the DCE scans in the pelvic region. In the past decade, several models have been recruited in pharmacokinetic analysis of clinical trial data and animal studies to calculate the plasma volume fraction, extravascular and extracellular volume fraction, and K^trans^ (42–45). However, few have examined whether the models are appropriate to the data (46, 47) and the variances and co-variances of parametric estimates, as well as the biases introduced by systematic errors, is generally lacking. Model selection, which is a potential solution since it defines the region of leaky microvasculature, a tumor signature, allows delineating different tumor regions and the temporal evolution of the local model and producing approximately unbiased estimate of vascular parameters that are relatively independent of variation in the details of image acquisition and equipment (48).

### Tumor Size and Location Are Important Considerations

Smaller lesions pose a challenge to using mpMRI to accurately and reproducibly target the DIL as imaging precision is known to become less accurate as volume decreases. This was previously reported by Groenendaal et al. (49), citing the impact of noise and geometrical distortions induced by MRI machines in complicating the validity of functional MRI techniques for smaller volumes. In this study, the mean contour volume for the T2W images, and the ADC and K^trans^ maps were 2.14 ± 2.1, 2.22 ± 2.2, and 1.84 ± 1.5 mL, respectively. It is reasonable to assume that with such small volumes, even slight differences in contours can result in significantly altered results.

The location of the DIL also plays a role in precise target delineation with mpMRI as lesions involving different zones of the prostate gland can present unique challenges. Prostate cancer most commonly involves the peripheral zone of the gland and appears as a region of homogenous low-signal intensity on T2W. Tumor involving of the central gland can be more difficult to discern (e.g., due to benign prostatic hyperplasia), but cross-observer consensus can be reached in up to 80% of cases (50). Similarly, K^trans^ does not reliably differentiate prostate cancer from benign prostatic hyperplasia within the central zone of the prostate gland due to similarities in microvascular density exhibited by both conditions. In fact, K^trans^ values were indistinguishable between tumor foci and the normal prostate gland for 19 patients in this study suggesting that the value of this mpMRI sequence may be limited to more peripheral lesions.

### Quantitative Correlation Between the DIL and Tumor Location

To evaluate the quantitative correlation between contours on each imaging modality and its statistical dependence on tumor location and Gleason grade group, the Hausdorff distance (HD), mean distance to agreement (MDA), Dice coefficient, and Jaccard index were calculated between the contours for each pair of MR sequences (i.e., T2W vs. ADC, T2W vs. K^trans^, and ADC vs. K^trans^). Table 2 summarizes the results of the statistical analysis based on tumor location. Between T2W-ADC and T2W-K^trans^, the Dice coefficient was 0.74 ± 0.13 and 0.61 ± 0.16, respectively, and the Jaccard index was 0.60 ± 0.15 and 0.46 ± 0.16, respectively. This suggests that there was a relatively high level of overlap between the contours regardless of tumor location, and that the contours were slightly more similar than they were divergent. Furthermore, the results were consistently better between T2W-ADC vs. T2W-K^trans^ which may reflect the fact that T2W images provide anatomical information whereas K^trans^ maps reflect the permeability of regional vasculature; consequently, although we expect to appreciate a certain level of correlation between the two MR sequences, it is understandable that a more substantial overlap between the contours was not observed. Conversely, the voxel-based Spearman correlation was 0.21 ± 0.18 and 0.13 ± 0.25, respectively, suggesting that the strength of the association between the contours was not very robust.

### Quantitative Correlation Between the DIL and Gleason Grade Group

Table 3 summarizes the results of the statistical analysis based on Gleason grade group. Between T2W-ADC and T2W-K^trans^, the overall Dice coefficient and Jaccard index were identical to the results based on tumor location. Furthermore, the voxel-based Spearman correlation between T2W-ADC was similarly low, especially for Gleason grade groups 4 and 5 (0.04 ± 0.19) suggesting a very poor correlation between anatomical imaging and diffusion-weighted and perfusion-based imaging in poorly-differentiated prostate cancer. Again, the results were consistently better between T2W-ADC vs. T2W-K^trans^.

### Incorporating a Boolean Sum Volume (BSV) to Better Delineate the DIL

As previously mentioned, the Spearman correlation between tumor location and Gleason grade group for the MR sequences was rather weak. This was particularly so between the T2W images and ADC maps for lesions with Gleason grade groups 4 and 5 (although lower Gleason grade group did not necessarily predict for a higher correlation). This data would suggest that constructing a BSV that incorporates T2W images and ADC maps may be reasonable for delineating the DIL on mpMRI, as the BSV would adequately represent radiographic disease that is both anatomically- and functionally-defined. This is supported by the fact that the level of correlation between T2W images and ADC maps was relatively high but far from reaching unity. This would, in theory, allow the entire DIL to be included in the radiation boost volume reducing the probability of a marginal miss especially with an adequately designed margin. The value of adding information provided by K^trans^ maps to the BSV remains investigational at this time as this mpMRI sequence was not reliably and consistently detectable as elaborated on above. A larger study population and a community consensus on quantitative analysis of K^trans^ may be warranted prior to its systematic incorporation into tumor delineation.

### Interobserver Variability

Nineteen cases were contoured by two radiation oncologists in an effort to assess for interobserver variability. There was a large difference in the Dice coefficient between the contoured DILs (23 and 19% for T2W-ADC and T2W-K^trans^, respectively). This is not surprising as significant interobserver variability is a known limitation in the interpretation of mpMRI images. As previously mentioned, the small volumes of the contours in this study (mean volumes ranging from 1.84 to 2.14 mL) may have amplified even the smallest of differences in tumor delineation, and whether these marginal statistical discrepancies would translate into meaningful differences in clinical outcome is debatable. Furthermore, it would be impractical for more than one radiation oncologist to delineate the DIL in clinical practice. A more pragmatic approach would be to develop an expert consensus guideline on DIL delineation coupled with suggestions for optimal clinical target volume margins to ensure adequate coverage.

### Contoured Pixel Values

The mean, minimum, and maximum contoured pixel values for ADC and K^trans^ are tabulated in Table 4. This information is intended as a baseline threshold recommendation for automatic segmentation of ADC and K^trans^ maps based on Gleason grade group. The contours used to obtain this data were delineated by the original physician on 90 patients. Of note, the K^trans^ mean pixel value is relatively high compared to reported tumor regions in previous studies (40, 51–53). Ktrans images used in this study were procured by a method explained in Huisman et al. (54), which results with differing pixel values than other commonly used methods. Since this study has shown that there is a large variation of ADC and K^trans^ values in each Gleason grade group, future work is needed to recommend specific thresholds for automatic delineation with the verification of whole-mount histopathologic section findings.

### Study Limitations

The limitations of this study include its retrospective design, inherent inconsistencies between functional MR images (e.g., different institutional imaging protocols such as contrast inject rate, variations in patient body mass index, and differences in spatial and temporal resolution), lack of histopathological validation, maximum b-value of 800 in calculating the ADC map, and tumor delineation by only two radiation oncologists. A prospectively designed study using standardized imaging with up-to-date protocols and contouring by a team of experienced radiation oncologists allowing for interobserver variability would strengthen the validity of these results.

## Conclusions

Using mpMRI to delineate a target volume for a radiation boost is an emerging area of interest and one that may improve clinical outcomes without increasing the toxicity associated with external beam radiation therapy. The intraprostatic lesions contoured on T2W images had a high level of agreement with those contoured on ADC maps, but there was little to no quantitative correlation of these results with tumor location and Gleason grade group. As shown in the study, there have been many technical hurdles to be solved for precision radiotherapy to target the tumor based on physiological imaging and understand its corresponding treatment outcome. A BSV incorporating all available MR sequences may be reasonable at the current stage in delineating the DIL boost volume for clinical practice. A larger study population and a community consensus on quantitative analysis of K^trans^ is warranted prior to its systematic incorporation into tumor delineation.

## Data Availability

The datasets generated for this study are available on request to the corresponding author.

## Ethics Statement

This study uses a public cohort. There is no need to request the approval from the IRB Committee.

## Author Contributions

JL is responsible for study design, implementation, manuscript writing. EC is responsible for statistical analysis. AF is responsible for the implementation. MP and ME is responsible for the study conception, implementation. NW is responsible for study conception, study design, and manuscript editing.

### Conflict of Interest Statement

The authors declare that the research was conducted in the absence of any commercial or financial relationships that could be construed as a potential conflict of interest.
